# Ontogenesis of Gonadotropin-Releasing Hormone Neurons: A Model for Hypothalamic Neuroendocrine Cell Development

**DOI:** 10.3389/fendo.2013.00089

**Published:** 2013-07-16

**Authors:** Erica L. Stevenson, Kristina M. Corella, Wilson C. J. Chung

**Affiliations:** ^1^Department of Biological Sciences, School of Biomedical Sciences, Kent State University, Kent, OH, USA

**Keywords:** GnRH, hypothalamus, fibroblast growth factor 8, embryonic and fetal development, neuroendocrine cells

## Abstract

The vertebrate hypothalamo–pituitary–gonadal axis is the anatomical framework responsible for reproductive competence and species propagation. Essential to the coordinated actions of this three-tiered biological system is the fact that the regulatory inputs ultimately converge on the gonadotropin-releasing hormone (GnRH) neuronal system, which in rodents primarily resides in the preoptic/hypothalamic region. In this short review we will focus on: (1) the general embryonic temporal and spatial development of the rodent GnRH neuronal system, (2) the origin(s) of GnRH neurons, and (3) which transcription – and growth factors have been found to be critical for GnRH neuronal ontogenesis and cellular fate-specification. Moreover, we ask the question whether the molecular and cellular mechanisms involved in GnRH neuronal development may also play a role in the development of other hypophyseal secreting neuroendocrine cells in the hypothalamus.

## Introduction

The ability of an organism to reproduce is of critical importance to the survival of not only the individual, but also of the species at large. In mammals, reproduction is under the tight regulation of a three-tiered body axis that consists of the neurons found in the preoptic area and hypothalamus, pituitary cells, and gonadal tissues, and therefore has been called the hypothalamo–pituitary–gonadal (HPG) axis. Functionally, gonadotropin-releasing hormone (GnRH) neurons are viewed as the most upstream regulatory component of the HPG axis. Most GnRH neurons are found around the very anterior tip of the third ventricle called the organum vasculosum lamina terminalis (OVLT) and their axons project to the external zone of the median eminence (ME) in order to release the decapeptide, GnRH, into the portal vein system. This in turn stimulates gonadotropin production and release from the gonadotrophs of the anterior pituitary into systemic circulation, ultimately causing steroidogenesis and gametogenesis in the gonads.

Key publications in the late 80s described in detail the neuroanatomical embryonic development of GnRH neurons in the rodent (1, 2). Based on the results of these landmark studies, a compelling argument has been made favoring that GnRH neurons originate (i.e., born) in the nasal compartment, at the level of the olfactory placode (OP), and migrate along the nasal septum to the preoptic region and hypothalamus by following the olfactory, vomeronasal, and terminal nerves (1, 3). Currently, the best estimates based on measurable GnRH peptide and mRNA levels suggest that this developmental process begins around embryonic day (E) 9.5, and is completed around E16.5 (1, 3).

Numerous studies have shown that the number of GnRH neurons in the mammalian brain is very limited. Indeed, the adult mouse and rat brain has a total of about 800–1000 GnRH neurons (4–7). Interestingly, there are studies reporting that those adult GnRH neurons are what remain from a larger pool of approximately 1900–2000 embryonic GnRH neurons found around E12.75 (7). These studies suggest that in addition to GnRH neuron path-finding, there is also an inherent selection process that takes place while GnRH neurons are migrating, which pares down the number of GnRH neurons in the adult rodent brain to 800–1000 total.

In this review, we will primarily focus on the central portion of the HPG axis; specifically, we will discuss in detail the mechanisms involved in GnRH neuronal ontogenesis and fate-specification. Moreover, we will discuss how this knowledge may be useful for understanding the embryonic development of other neuroendocrine cell types that have been found in the preoptic region and the hypothalamus.

## Presumptive Origins GnRH Neurons

### Olfactory placode

As previously alluded to, GnRH neurons are first found outside the central nervous system (CNS), even though the CNS is their final residence. Using immunocytochemistry and *in situ* hybridization, two landmark studies showed that GnRH expressing cells could be found only in a specific part of the nasal compartment, the OP, on E11.5 from which they migrate to the forebrain (1, 3).

Interestingly, proliferation studies showed that the vast majority (∼80%) of mouse GnRH neurons undergo their last division in the medial–ventral OP between E9.5 and E10.5 (2). These data suggest that the anatomical localization of GnRH progenitor cell ontogenesis is localized in the embryonic medial–ventral OP. Interestingly, these post-mitotic GnRH progenitor cells do not express detectable levels of GnRH mRNA or peptide prior to E11.5 (1, 2). In this sense, one can define the period of GnRH neuronal fate-specification to occur between E9.5 and E11.5. The absence of measurable GnRH peptide prior to E11.5 is a major hurdle for studying the molecular processes that regulate GnRH neuronal fate-specification because, at present, the only marker for GnRH neurons is the ability to express the GnRH peptide itself. Therefore, post-mitotic OP progenitor cells that are destined to become GnRH neurons cannot be identified during a large portion of fate-specification, before they express measurable GnRH peptide levels. Taken together, these studies make a strong case supporting the central hypothesis that GnRH progenitor cells are born, and become post-mitotic in the medial–ventral OP.

Further support for this hypothesis comes from the observation that humans who are deficient in GnRH signaling not only exhibit hypogonadotropic hypogonadism, which causes infertility, but are also completely or partially lacking the ability to smell (i.e., anosmia or hyposmia) (8–11). One such reproductive disorder in humans is known as Kallmann syndrome (KS) (12–16). The presence of olfactory function defects in KS patients is in line with the observation that the presumably “newly” born GnRH progenitor cells are first detected in the medial–ventral OP.

The OP is an ectodermal region that gives rise to both non-sensory respiratory epithelium and sensory olfactory epithelium. The olfactory epithelium eventually develops into the main olfactory and vomeronasal systems (17, 18). Ablation studies have provided supporting evidence for the OP being the birthplace of GnRH neurons, albeit sometimes ambiguously. For example, amphibians that undergo OP removal ultimately lack the olfactory epithelium, nerve, and bulb, as well as, GnRH neurons of the forebrain (19, 20). Interestingly, when the OP is removed in rat and chick embryos it does not result in total loss of forebrain GnRH neurons. Rather, small populations of GnRH neurons are found in the septum of rats, suggesting that these did not arise from the OP (21, 22). However, it has been argued that the method of ablation may have allowed some of the OP cells to survive, consequently allowing the emergence of a limited population of GnRH neurons to migrate to the septum.

Studies in chicks further pinpointed that forebrain GnRH neurons are eliminated only when the respiratory epithelium is ablated, and not the olfactory epithelium (23). Conversely, ablation of the olfactory epithelium does not affect the development of the GnRH neuronal system in chicks (24, 25). Nonetheless, only a small percentage of GnRH neurons have been found migrating from the respiratory epithelium of normal chicks, whilst the majority of GnRH neurons are from the olfactory epithelium (26). Studies in mice have shown that GnRH neurons are not exclusively localized in the olfactory epithelium, but can also be found in the respiratory epithelium (27) as delineated using the transcription factor marker activator protein 2α (AP2α). Moreover, AP2α is reported to be co-localized in some of these newly formed GnRH neurons (27). In the same study, a number of GnRH neurons were found to be positive for nestin, which is another marker of the respiratory epithelium. In contrast, some GnRH neurons do not express olfactory epithelium markers, such as *Mash-1*, *Math4A*, *Math4C*/*neurogenin1*, and *NeuroD* (27). Taken together, these data demonstrate that both the respiratory and olfactory epithelium may contribute significantly to the OP’s ability to generate GnRH neurons. This conclusion, along with the inability to directly follow GnRH progenitor cells from their birth (due to lack of a marker for GnRH neurons other than the GnRH peptide itself) has led to a proposed alternative birth place of GnRH neurons other than the olfactory epithelium in the OP (see below).

### Neural crest

Studies of the GnRH neuronal system in zebrafish have cast increasing doubt on the accepted paradigm that GnRH neurons originate solely from within the olfactory epithelium in the medial–ventral OP. For example, analysis of two knockout zebrafish strains, *you-too* and *detour*, showed that not only is the pituitary lacking or reduced, but there is also a concomitant loss of hypothalamic GnRH neurons (28, 29). Interestingly, these animals have normal olfactory organ development suggesting that the loss of GnRH neurons cannot be due to the loss of the OP. This inconsistency in GnRH neuronal origin may be due to species differences. Indeed, mouse homologs of the aforementioned zebrafish pituitary knockouts do not show a loss of GnRH neurons (30–33). Therefore, we can infer that GnRH progenitor cells are not likely to originate from the developmental tissues that give rise to the anterior pituitary in mammals.

In several vertebrate species the neural crest has been implicated as a possible contributor to the formation of the OP (34–36). This region arises from the edge of the neural plate and shares a border with the region that eventually becomes the OP. Neural crest cells have been shown to migrate toward the presumptive OP, and therefore, are likely to have contributed specific cell populations to the developing OP (37). Conclusive evidence supporting this inference comes from zebrafish studies where premigratory neural crest cells were labeled with lysinated rhodamine dextran *in vivo* (38, 39). Subsequent double-labeling studies showed that a subset of the tagged neural crest cells expressed GnRH mRNA and peptide during the migratory phase of GnRH neuronal development (29).

These original data have been recently replicated in mice. Indeed, a small proportion (i.e., ∼30%) of the GnRH neurons found in the OP had a genetic lineage similar to cells that arise in the neural crest (35, 40). However, the other 70% of the total population of GnRH neurons originated from placodal ectoderm progenitors. In contrast, recent cell fate tracing studies in chicks determined that all GnRH progenitors originate from ectodermal placodes, suggesting that the developing neural crest does not contribute to the emergence of GnRH progenitors in this species (41). In short, GnRH neurons are likely to originate from both the neural crest and OP. However, the degree to which each of these two proliferative zones contributes to the GnRH neuronal system may be species-dependent.

## Genes Regulating Neuroendocrine Cell Development

The anatomical development of hypothalamic neuroendocrine cells has been studied in much detail since the late 70s. For instance, Altman and Bayer showed that hypothalamic cells are primarily born during the second half of rat gestation, and originate from the neuroepithelium of the fetal third ventricle (42). Since then, many studies have contributed to the elucidation of the underlying molecular mechanisms of hypothalamic morphogenesis.

Unlike other hypothalamic neuroendocrine cells, the origins of GnRH neurons are extra-hypothalamic; however, the development of the GnRH neuronal system is arguably one of the best-studied neuroendocrine cell systems within the hypothalamus. Consequently, it is possible that some of the molecular factors that have been found to regulate GnRH neuronal development in the nasal and forebrain compartments may also be relevant, and involved in the embryonic development of non-GnRH hypothalamic neuroendocrine cells, such as those that express vasopressin and oxytocin.

Due to major advances in molecular techniques over the last couple of decades it has become possible to genetically screen individuals with GnRH deficiencies. For example, KS patients have been extensively screened in order to study whether they harbor specific gene mutation(s) that may have led to their infertility and olfactory deficits. These studies have identified many factors regulating fate-specification and migration of GnRH neurons [as reviewed in (43–47)]. Some of these factors and their contributions are described below.

The first mutated gene to be found in KS patients was anosmin-1 (as described below) (48–50). Following genetic screening, studies found further evidence supporting the idea that KS may be the result of a myriad of mutated genes. Indeed, KS patients have been shown to harbor hemizygous mutations for fibroblast growth factor receptor-1 (*Fgfr1*) (13, 51, 52), fibroblast growth factor 8 (*Fgf8*) (14, 15, 53), prokineticin 2 (*Prok2*) (54) and its receptor (*Prokr2*) (55), and chromodomain helicase-DNA-binding 7 (*Chd7*) (56–58). These studies have been instrumental in providing possible candidate genes that may play a role in GnRH progenitor cell fate-specification, and possibly provide further insight into the origin of GnRH neurons.

### Anosmin-1

Studies in humans revealed that *KAL-1* mutations underlie the X-linked form of KS in humans (13, 48, 49, 59, 60). *KAL-1* (also known as *Anosmin-1*) encodes an extracellular matrix protein that is secreted by the cranial neural crest, a developmental region in mice from which approximately 30% of the GnRH progenitors originate, and provides autocrine regulation of neural crest formation in chicks (61). Anosmin-1 plays a role in the regulation of GnRH neuronal migration in rats and humans by promoting the formation of the lateral olfactory tract and the collateral branches of the mitral and tufted cells toward the olfactory cortex (13, 62–65). It does this, in part, by promoting FGF signaling (66).

There is evidence that anosmin-1 expressing cells are readily found within the hypothalamus in rats and zebrafish (67, 68). Interestingly, recent studies have identified the presence of anosmin-1 protein within bipolar cells in the most rostral hypothalamic neuroepithelium that lines the third ventricle in rat embryos (67), which has been shown to be the proliferative zone that gives rise to many, if not all, hypothalamic neurons (42). Furthermore, it has been reported that these hypothalamic neuroepithelia anosmin-1 positive cells are not detected in the same region in postnatal rats. Currently, the identity and the function of these anosmin-1 positive cells are unknown. Based on its function during GnRH neuron migration, it may be speculated that these anosmin-1 cells facilitate radial or tangential neuroendocrine cell migration from the proliferative zone of the third ventricle.

### Fibroblast growth factor receptor-1 and fibroblast growth factor 8

Studies in humans and rodents have cemented the concept that FGF signaling is critically important for GnRH progenitor cell birth and proliferation (15, 16, 69, 70). Moreover, loss-of-function mutations in both *Fgfr1* and *Fgf8* have been detected in KS patients (15, 51). Our basic studies using homozygous *Fgfr1* and *Fgf8* hypomorphic newborn mice showed that the GnRH neuronal system was reduced (i.e., ∼90%) or completely absent, respectively (14, 15). Moreover, the GnRH neuronal population in the heterozygous *Fgfr1* and *Fgf8* hypomorphs was also significantly reduced as compared to the wildtype newborns (14, 15). Following studies furthermore found evidence that the reduced GnRH neuronal system in these hypomorphic mice causes abnormal reproductive function (71). Together, these results provided a fundamental explanation for the reproductive defects found in KS patients who harbor *Fgfr1* and/or *Fgf8* mutations.

The elimination of GnRH neurons likely occurred during the emergence phase of GnRH neuronal development (i.e., ∼E9.5–E11.5). In contrast to wildtype E11.5 embryos, no GnRH neurons were detected in the medial–ventral OP of homozygous *Fgf8* hypomorphs (14). Currently, it is unknown whether the elimination of the GnRH progenitor cells is the cause of abrogated FGF8-dependent proliferation or cell survival. However, circumstantial evidence favors the second option given that the presence of increased apoptosis has been reported for the E10.5 OP (70).

Subsequent studies in non-GnRH neuroendocrine systems in the mouse hypothalamus showed that FGF signaling is also involved in the development of hypothalamic neuroendocrine cells that express kisspeptin, vasopressin, and oxytocin (70–73). Specifically, the number of kisspeptin neurons in the adult heterozygous hypomorphic *Fgfr1* and *Fgf8* compound mice was significantly higher than wildtype mice (71). Currently, it is unknown whether the increase in kisspeptin neurons in these compound *Fgfr1*/*Fgf8* mice is the result of increased proliferation or survival of kisspeptin neurons during embryonic development. Alternatively, a more parsimonious explanation could be that the kisspeptin system has been “ramped” up as a compensatory mechanism to counter the reduction in the number of GnRH neurons in these compound *Fgfr1*/*Fgf8* hypomorphic mice.

It should be noted that FGF signaling may play a role in maintaining neurochemical phenotype/identity. Indeed, Brooks and colleagues recently reported a major reduction in the number of oxytocin neurons in the paraventricular nucleus (PVN) and supraoptic nucleus (SON) of the hypothalamus (72). Upon further examination, an interesting divergent role of FGF8 signaling during oxytocin neuronal development was detected. Indeed, while the loss of oxytocin neurons in the SON was correlated with a loss in the oxyphysin–prohormone levels, this was not the case for the PVN oxytocin neurons (72). These data indicate that FGF8 signaling plays a role in shaping the hypothalamic oxytocin system on two levels: anatomy (i.e., cell number) and cellular neuropeptide processing.

The hypothalamic vasopressinergic system was also highly compromised in heterozygous and homozygous *Fgf8* hypomorphic newborn mice (73). Specifically, the number of vasopressin neurons was found to be reduced in the PVN, SON, and suprachiasmatic nucleus (SCN) (73). Interestingly, the percentage of vasopressin neuronal loss was much higher in the SCN than in the PVN suggesting a ventral–dorsal gradient in FGF8-dependence. These results are in line with previous studies showing that *Fgf8* mRNA expression in the E13.5 mouse embryo is highest in the ventral zone of the developing hypothalamus, which diminishes dorsally (Allen Developing Mouse Brain Atlas). Taken together, these studies support the proposition that the ventral hypothalamic neuroendocrine cells (i.e., vasopressin or oxytocin) are more dependent, and hence, vulnerable to disruptions in FGF8 signaling during embryonic development.

### Prokineticin 2/Prokineticin receptor 2

In contrast to the FGF signaling system, Prok2 (74) and its G-protein coupled receptor (Prokr2) (75–77) were shown to primarily regulate GnRH neuron migration and neuroendocrine function in *Prok2* and *Prokr2* null mutant mouse studies (78–80), which may underlie the abnormal or absent pubertal onset found in humans (54, 80).

Prokineticin 2 null animals display a greatly reduced number of GnRH neurons in the medial preoptic area, and 50% of them show asymmetric olfactory bulb neurogenesis (78, 80). The loss of medial preoptic GnRH neurons is likely due to the malformation of the olfactory vomeronasal axonal pathways (79). Humans with mutations in *Prok2* or *Prokr2* present with either KS or normosmic hypogonadotropic hypogonadism (54, 55, 81). Interestingly, Prok2 and Prokr2 are not localized in GnRH neurons (80). Due to this fact, it has been suggested that Prok2/Prokr2 impact GnRH migration by acting on the fibers that guide GnRH neurons into the forebrain (82).

Both Prok2 and Prokr2 expression has been localized within several nuclei of the hypothalamus, most notably in the mouse SCN and PVN (83). Indeed, *Prokr2* null mice have disruptions in circadian activity and thermoregulation, which are two major functions of the hypothalamus (84). Thus far, it is unknown whether the loss of *Prokr2* expression causes developmental defects within the embryonic organization of the SCN. However, it is known that *Prokr2* null mice exhibit defective neuronal progenitor proliferation and migration from the subventricular zone, which is likely to underlie the decreased volume of the olfactory bulbs (85). This defect was further compounded by the increased levels of olfactory bulb apoptosis (85). Taken together, these data favor the possibility that Prok2 and Prokr2 may also be involved in the embryonic organization of hypothalamic neuroendocrine cells.

### Chromodomain helicase-DNA-binding 7

Chromodomain helicase-DNA-binding 7 mutations have been causally linked to the main cause of CHARGE syndrome, a disorder that is characterized by a number of symptoms, including heart defects, ocular coloboma, genital hypoplasia, and KS (86). Mouse studies have shown that *Chd7* expression is present in the OP from E10.5 onward, which could explain why CHARGE syndrome patients manifest anosmia and hypogonadotropin hypogonadism (56). In line with these clinical observations, *Chd7* null mice have compromised reproductive fitness and significantly less GnRH neurons (87). The reduction of GnRH neurons may be linked to the lower levels of cellular proliferation in the E10.5 OP (87). Currently, there are no studies implicating that *Chd7* might also play a role in the development of non-GnRH hypothalamic neuroendocrine cells.

### Pax

The PAX family of transcription factors contains a unique DNA binding sequence known as the paired-box, a DNA binding homeo-domain (88, 89). To date, there have been 9 *Pax* genes isolated and all but PAX-1 have been localized in the developing CNS (89). This family has been described to be involved in early animal development and tissue specification.

*Pax-6* is of special interest because of its importance during the development of the eye, olfactory system, and forebrain. Mice with a semi-dominant point mutation in the *Pax-6* gene are known as small-eye (*Sey*) (90, 91). *Sey* homozygotes do not develop eyes or an OP, and do not survive to birth (92, 93). In mice, *Pax-6* expression can be detected in mice on E8.5 in the eye, olfactory system, pituitary, brain, and spinal cord (94–96). Furthermore, PAX-6 is localized in the neocortex, lateral ganglionic eminence, thalamus, and hypothalamus (97).

*Sey* heterozygotes survive to postpartum, but suffer varying degrees of eye malformations (92, 98, 99). Since *Sey* mice do not form OPs they have been useful models for examining the origins of GnRH neurons. Indeed, *Sey* homozygotes completely lack GnRH neurons in all regions of the brain (100), which provides further evidence supporting the medial–ventral OP as the birth place of GnRH progenitor cells. Of course, the absence of OP development may also have been detrimental to GnRH progenitor cells that are of neural crest origin (35).

No direct evidence has been reported about the role of PAX-6 during the hypothalamic neuroendocrine development. However, mapping studies have shown that *Pax-6* expression can be found in the subventricular neuroepithelium cells of the third ventricle in the E14.5 mouse hypothalamus (97, 101). Furthermore, anatomical studies in *Sey* mice have reported that the compartmentalization of the embryonic hypothalamus is disrupted causing it to expand beyond its normal boundaries (101). Furthermore, tracing studies have shown that the mammillothalamic tract is abnormal in *Sey* null mouse hypothalamus (102). Therefore, it is possible that *Pax-6* may be important for the emergence of non-GnRH hypothalamic neuroendocrine cells, although this has not yet been studied in great detail.

### Sox

The *Sox* gene family belongs to the high mobility group (HMG) superfamily (103). These proteins bind to the minor groove of DNA in a highly sequence-specific manner (104–106). There have been at least 20 *Sox* genes identified, which are categorized into eight classes (A–H). Although the Class B *Sox* genes are known to be involved in embryonic neuronal development (107) and differentiation of the neuroepithelium (108), it is the Class C *Sox* genes that provide evidence of a role in the regulation of GnRH expression. There are two SOX-binding sites located on the intron A region of *GnRH*, which possesses a putative transcriptional enhancer. When tested, only SOX-4 and SOX-11 (members of Class C) dramatically increased luciferase reporter activity (109). Both SOX-4 and SOX-11 were localized within ∼80% of GnRH neurons and found in significantly lower abundance in non-GnRH hypothalamic cells (109). To our knowledge, there are no data available describing the role of SOX-4 or SOX-11 during hypothalamic neuroendocrine cell development.

## Conclusion

In this short, and by no means exhaustive review we argue that the molecular mechanisms involved during GnRH neuronal fetal development may also provide some insights into the development of non-GnRH hypothalamic neuroendocrine cells. In general this approach has been fruitful in elucidating some aspects of the molecular underpinning of neuroendocrine cells during their fetal development. As such, a promising and well-described candidate seems to be FGF8. Indeed, mice that are hypomorphic for *Fgf8* exhibit dramatic GnRH and non-GnRH hypothalamic neuroendocrine cell defects (see above). However, there are still many questions that arise from these observations. For instance, do these factors affect the proliferation, fate-specification, migration, and/or survival of hypothalamic neuroepithelium that are destined to become part of the various neuroendocrine systems in the hypothalamus? Moreover, do other members of the reviewed signaling systems also play a role in the development of the hypothalamus? The direct examination of these types of questions will be beneficial in order to better understand the fetal organization of the hypothalamic neuroendocrine systems.

## Conflict of Interest Statement

The authors declare that the research was conducted in the absence of any commercial or financial relationships that could be construed as a potential conflict of interest.
